# Recognition of Wheat Leaf Diseases Using Lightweight Convolutional Neural Networks against Complex Backgrounds

**DOI:** 10.3390/life13112125

**Published:** 2023-10-26

**Authors:** Xiaojie Wen, Minghao Zeng, Jing Chen, Muzaipaer Maimaiti, Qi Liu

**Affiliations:** 1 Key Laboratory of the Pest Monitoring and Safety Control of Crops and Forests of the Xinjiang Uygur Autonomous Region, College of Agronomy, Xinjiang Agricultural University, Urumqi 830052, China; adgerx@163.com (X.W.); xjauzeng@163.com (M.Z.); muzappar0829@163.com (M.M.); 2 Key Laboratory of Prevention and Control of Invasive Alien Species in Agriculture & Forestry of the North-Western Desert Oasis, Ministry of Agriculture and Rural Affairs, Urumqi 830052, China

**Keywords:** convolutional neural network, training strategy, initial learning rate, wheat leaf diseases

## Abstract

Wheat leaf diseases are considered to be the foremost threat to wheat yield. In the realm of crop disease detection, convolutional neural networks (CNNs) have emerged as important tools. The training strategy and the initial learning rate are key factors that impact the performance and training speed of the model in CNNs. This study employed six training strategies, including Adam, SGD, Adam + StepLR, SGD + StepLR, Warm-up + Cosine annealing + SGD, Warm-up + Cosine, and annealing + Adam, with three initial learning rates (0.05, 0.01, and 0.001). Using the wheat stripe rust, wheat powdery mildew, and healthy wheat datasets, five lightweight CNN models, namely MobileNetV3, ShuffleNetV2, GhostNet, MnasNet, and EfficientNetV2, were evaluated. The results showed that upon combining the SGD + StepLR with the initial learning rate of 0.001, the MnasNet obtained the highest recognition accuracy of 98.65%. The accuracy increased by 1.1% as compared to that obtained with the training strategy with a fixed learning rate, and the size of the parameters was only 19.09 M. The above results indicated that the MnasNet was appropriate for porting to the mobile terminal and efficient for automatically identifying wheat leaf diseases.

## 1. Introduction

Wheat, the most crucial cereal crop, is directly intertwined with the survival and advancement of humanity [1]. Wheat leaf diseases significantly impact the secure production of wheat. Common wheat foliar diseases include wheat rust [2], wheat powdery mildew [3], wheat scab [4], and so on. In the past, expert diagnosis was the primary way to identify plant diseases [5]. This approach placed excessive reliance on the subjective expertise of pathologists and was susceptible to subjective and varying opinions. Moreover, the diagnostic scope was limited, demanding significant labor efforts. With the development of technology, modern molecular biology techniques have since been widely used in pathogen analysis [6]. These techniques boast a high accuracy rate yet necessitate a prolonged operational procedure and consume considerable time. Unfortunately, these techniques cannot be employed for real-time onsite detection, and their widespread adoption poses significant challenges. Therefore, the automated and rapid identification of wheat diseases on a large scale holds immense importance for advancing the development of secure wheat production in the future.

In recent years, deep learning (DL) has been successfully applied to the field of intelligent agriculture, including areas such as pest detection [7], plant and fruit identification [8], crop and weed detection, and classification [9,10]. Deep learning exhibits a greater capacity to extract features from agricultural images and structured data compared to traditional machine learning. Additionally, it has the capability to integrate modern technology and equipment, facilitating the rapid advancement of smart agriculture and accelerating the transition from conventional farming to smart agriculture.

Dong [11] proposed a differential amplified convolutional neural network (DACNN) that was used to recognize images of wheat leaf disease, and the average recognition accuracy was 95.18%. Genaev [12] used a method to identify five fungal diseases of wheat sprouts that could be used to identify both individual and multiple diseases. A technique based on an image hash algorithm was employed to create the datasets in order to lessen the degradation of the training data. The disease recognition system, which was built on an EfficientNet-based convolutional neural network (CNN), had a best accuracy of 94.2%. In order to identify wheat yellow rust, Pan [13] applied the high-precision classification results of traditional algorithms as weak samples and used the pyramid scene parsing network (PSPNet) semantic segmentation model to distinguish between healthy wheat and yellow rust wheat using bare soil in small-scale UAV images. The classification results showed that the recognition accuracy of the PSPNet model reached 98%. Goyal [14] developed a novel deep learning model to classify wheat diseases with an accuracy of 97.88%. Compared with VGG16 and ResNet50, its accuracy increased by 7.01% and 15.92%, respectively. Jiang [15] used field images to identify diseases for seven typical models based on different training strategies, and the transfer learning method that readjusted all parameters exhibited the highest accuracy. Using this method on the test dataset, Inception-v3 achieved the highest recognition accuracy of 92.5%. Pan [16] proposed a method for identifying wheat rust based on ensemble learning (WR-EL) and enhanced the stochastic gradient descent with the warm restarts algorithm (SGDR-S). The recognition accuracy of WR-EL increased by 32%, 19%, 15%, 11%, and 8%, respectively, when compared with five CNN models, namely VGG, ResNet 101, ResNet 152, DenseNet 169, and DenseNet 201. Nigam [17] utilized the fine-tuning model based on the EfficientNet architecture to identify wheat rust and confirmed that the EfficientNet B4 model with fine-tuning had the best accuracy of 99.35% and was suitable for usage on mobile devices.

In deep learning, the learning rate is one of the most important hyper-parameters. In order to ensure the performance and convergence speed of the model, it is necessary to choose a suitable learning rate [18]. In choosing a learning rate, some challenges have to be faced. For example, due to the suitable learning rate being different, it is difficult to evaluate the advantages and disadvantages of different models with the same parameters. Moreover, the performance of a model is closely related to its previous learning rate scheduling strategy because the current performance is the result of the cumulative influence of all learning rates used during training, and the changing of the dataset and model structure could also lead to a large discrepancy in model performance at the same learning rate [19]. With the development of a training strategy and the optimizer, the learning rate has changed from a fixed learning rate to a scheduled learning rate [20,21,22,23]. The optimizer could adjust the learning rate adaptively to improve the recognition effect of the model. However, more parameters may need to be adjusted, such as the parameters in the optimizer that could also affect the training effect. At present, cyclic cosine attenuation [24], gradient descent [25], and warm-up training [26] are the most popular options; however, these methods do not guarantee an improved performance at certain learning rates.

Coleman [27] explored the effects of different initial learning rates and optimizers on model performance; the results showed that the different optimizations interacted to a certain extent. When the learning rate was combined with the optimizer, it could accelerate the convergence and slightly improve the accuracy of the model. Wang [28] explored the effects of different learning rates (10^−3^, 10^−4^, and 10^−5^), different batch sizes (8, 16, and 32), and data enhancement on the performance of the model in the field of maize disease recognition. The results showed that when the learning rate was set to 0.0001, compared with LeNet, AlexNet, and GoogLeNet, the improved model had a better effect on disease recognition. Fan [29] explored the influence of VGG16 models with different learning rates on the detection and classification of ginkgo biloba embryos, and the results showed that the overall performance of the model decreased with the order of learning rates 10^−4^, 10^−5^, 10^−6^, and 10^−3^, and it was difficult to fully train the fixed learning rate. In this case, the gradient attenuation strategy with an update period of 4 and an attenuation coefficient of 0.1 were used to achieve the lowest loss and highest accuracy, indicating that the learning rate attenuation strategy was feasible. Fan [30] compared the effects of different optimizers (Adam and SGD) and initial learning rates on model performance. It was found that the model performance was better when the learning rate was 10^−3^ under the same training mode than when the learning rate was 10^−2^, and the Adam optimizer had a better performance under the same learning rate. The average accuracy of grape leaf disease identification was 98.02%. Liu [31] used the SGD optimizer to explore the performance of different learning rate scheduling methods on different datasets, and the results showed that learning rate attenuation was crucial for model optimization. In the process of model optimization, the learning rate should not be attenuated too quickly. When the stochastic gradient descent with warm restarts (SGDR) method is applied, the learning rate cycle should not be too small, and a larger cycle can be used in the later stage of model training to avoid the failure of optimization caused by the learning rate fading too fast in one direction and the late update of the optimizer. Hideaki [32] dealt with nonconvex stochastic optimization problems in deep learning and provided an appropriate learning rate based on the theory that the adaptive learning rate optimization algorithms such as Adam and AMSGrad could approach the stationary point of the problem. Experiments showed that algorithms with a constant learning rate performed better than algorithms with a decreasing learning rate.

The aforementioned studies’ findings supported the CNN model’s advantages in identifying crop diseases, which has good application prospects in wheat disease recognition. However, due to the different initial learning rates and the diverse training methodologies employed by various models, differences in recognition accuracy emerged. Therefore, the objective of this study was to delve into the impact of training strategies and initial learning rates on the model’s performance. A lightweight CNN suitable for wheat leaf disease was designed. In particular, models such as MnasNet and EfficientNetV2 are rarely used for agricultural disease identification at present. This study was organized into four main sections as follows:

(1) In order to closely simulate the authentic wheat environment, a dataset of two wheat leaf diseases (wheat powdery mildew and wheat stripe rust) and healthy leaves with intricate natural backgrounds was constructed.
(2) Five lightweight neural network models were fine-tuned using pre-trained weights from the ImageNet dataset. The influence of various initial learning rates on model performance during the training process was discussed.
(3) The effects of six different training strategies on the five models was evaluated.
(4) The optimal initial learning rate and training strategy were selected to retrain the MnasNet model, and the model’s performance advantages were confirmed by visually verifying the model results.

## 2. Materials and Methods

### 2.1. Image Acquisition

**Figure 1:**
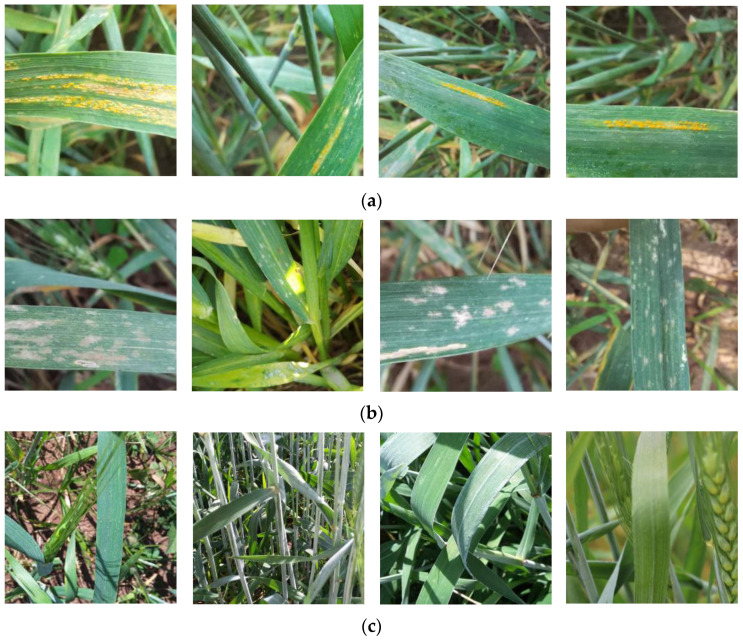
Examples from the wheat disease dataset: (**a**) wheat stripe rust; (**b**) wheat powdery mildew; (**c**) healthy wheat.

In this study, the images of wheat stripe rust and powdery mildew as well as healthy wheat leaves were obtained through field shooting and network acquisition, which were used to create datasets. The images from field shooting were manually captured with a 48-megapixel mobile phone in the open environment with a complex background of natural light during the 2022–2023 period in the Ili Kazakh Autonomous Prefecture and the Bayingoleng Mongolian Autonomous Prefecture, Xinjiang, which are the main wheat-producing regions. The wheat in these sampled areas is grown according to local conventional cropping patterns. Each image was manually annotated after being examined by a phytopathologist. The raw datasets comprised 1350 images of wheat stripe rust, powdery mildew, and healthy wheat leaves (Figure 1).

### 2.2. Image Preprocessing

**Figure 2:**
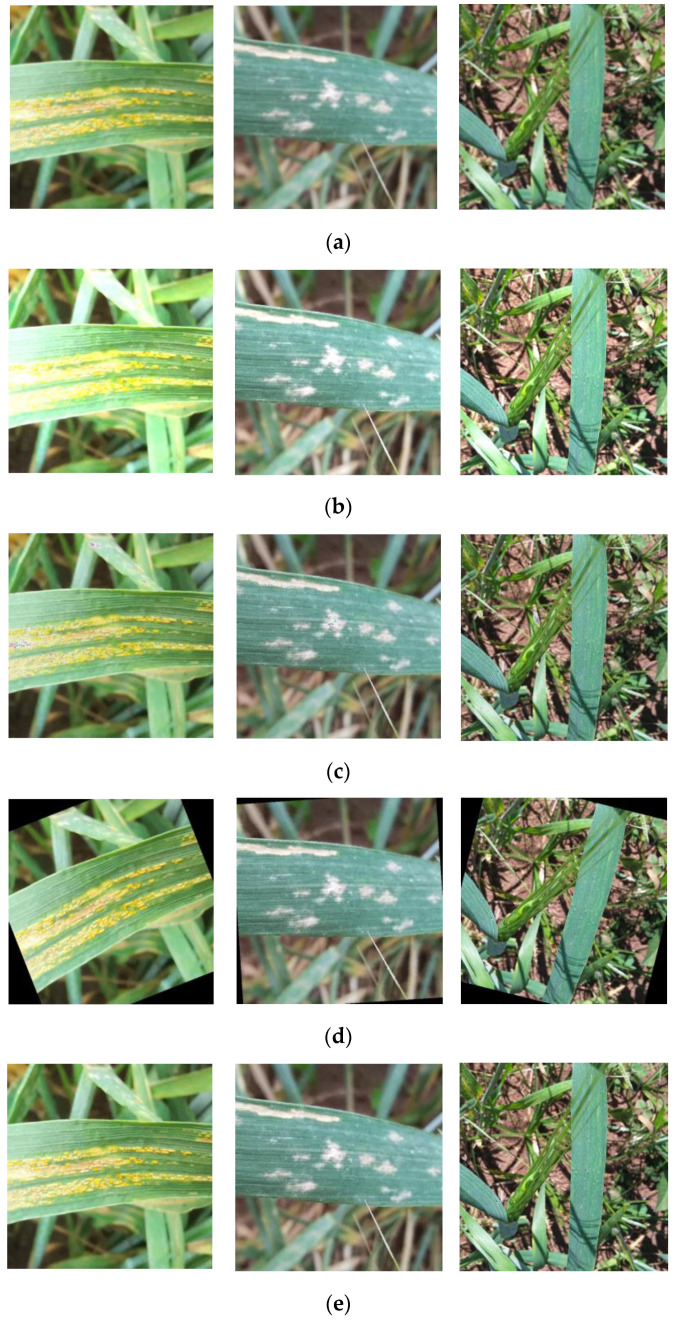
Partially augmented images obtained using five methods: (**a**) mosaic blur; (**b**) random brightness; (**c**) Gaussian noise; (**d**) random rotation; (**e**) random scaling.

In deep learning, the larger the sample size, the better the training effect and robustness of the model [33]. For this reason, all the raw data underwent augmentation, including rotation, scaling, brightness adjustment, mosaic blur, and Gaussian noise, which were processed using the Image, ImageFilter, and ImageEnhance modules of Python’s PIL library (Figure 2). After data augmentation, a total of 2700 images were obtained, and the augmentation dataset was divided into a training set, a validation set, and a test set at a ratio of 7:2:1 [34]. The distribution of sample numbers in the dataset is shown in Table 1. Before initiating learning training by the 5 different models, the images were scaled to 224 × 224 pixels using the transform command and normalized to [0,1] to lessen model overfitting and hasten model convergence.

### 2.3. Lightweight Convolutional Neural Networks

This study used 5 representative lightweight convolutional neural networks to identify wheat disease, namely MobileNetV3, ShuffleNetV2, GhostNet, MnasNet, and EfficientNetV2. The details of these lightweight CNNs are as follows.

#### 2.3.1. MobileNetV3-Large

MobileNetV3, which was suggested in 2019, has two forms—large and small—to accommodate high-resource and low-resource model training, respectively [35]. A novel efficient segmentation decoder termed lite reduced atrous spatial pyramid pooling (LR-ASPP) was proposed. On the basis of the V2 series, the block module was updated in the model design, and the attention mechanism squeeze excitation (SE) module was added. By reorganizing the structure of the time-consuming layer using network architecture search (NAS) search, the 32 convolution kernels of the first convolution layer were reduced to 16, simplifying the last stage. The swish activation function was revised, which evolved from the ReLU activation function, thus creating the h-swish activation function. In terms of recognition accuracy, latency reduction, and detection speed, MobileNetV3 improved the performance of classification, detection, and segmentation to a greater extent than its MobileNet family ancestors.

#### 2.3.2. ShuffleNetV2

ShuffleNetV2 was proposed in 2018 with the goal of improving computing performance and accuracy [36]. In comparison to ShuffleNetV1, two new designs for channel rearrangement and group convolution were implemented to minimize computation and parameters. Channel rearrangement was one of the core concepts of ShuffleNetV2. It entailed partitioning the input feature map into channels, performing convolution computations on each feature group, and ultimately reconfiguring separate features based on specific regulations. This approach led to an enhancement in feature diversity and expressive capability. The parameter amounts were subsequently reduced via group convolution. The input channel was divided into numerous groups through group convolution, then an independent convolution operation was executed in each group. By reducing the number of parameters in the convolution kernel, the model’s complexity was decreased. Via channel rearrangement, the information between various groups was able to be concurrently communicated, which retained the model’s expressiveness. Finally, additional optimization techniques were used by ShuffleNetV2, such as upsampling and 1 × 1 convolution for dimensionality reduction, which further minimized the model’s computation requirements.

#### 2.3.3. GhostNet

GhostNet was first proposed in 2019 [37] and primarily included two essential technologies: Ghost Module and Ghost Bottleneck. Ghost Module was used to separate the input channel into the “main channel” and the “ghost channel” and then splice the two channels’ outputs together, which was able to minimize the number of calculations and parameters while retaining a high level of feature expression. GhostNet’s most fundamental building block was the Ghost Bottleneck. The bottleneck structure in ResNet was improved by Ghost Bottlenecks by way of a combination of 1 × 1, 3 × 3, and 1 × 1 convolution kernels. In particular, the 3 × 3 convolution kernel was only calculated in the main channel, while the ghost channel directly passed through the main channel. GhostNet used 16 Ghost bottlenecks for stacking. When the resources were limited, GhostNet still exhibited a better performance that significantly reduced the computational complexity and the number of parameters while maintaining high feature-expression capabilities.

#### 2.3.4. MnasNet

In 2018, MnasNet was proposed, and its architecture aimed to minimize computation and parameters while retaining accuracy [38]. An automated search technique was used in MnasNet to create the model structure. The network search algorithm was utilized to automatically explore a diverse range of potential model structures by selecting an optimal combination from convolutional layers, expanded convolutional layers, pooling layers, and other modules for network construction. Additionally, each module’s hyper-parameters were determined by the search algorithm automatically, facilitating the rapid establishment of an effective network structure in MnasNet. Moreover, MnasNet incorporates a platform-aware approach to enable model tuning for diverse hardware platforms. The network structure and hyper-parameters can be automatically modified based on varying computational resources and memory constraints, ensuring optimal performance across different devices.

#### 2.3.5. EfficientNetV2

In 2021, a new effective deep learning model called EfficientNetv2 was released by Google [39]. Its algorithm differed from its forerunner in two key ways. Firstly, it employed neural architecture search (NAS), a new search space that incorporated a compound scaling algorithm that considered the training set size along with network width, depth, and resolution. Secondly, it utilized progressive learning to gradually increase the network’s width and depth while dynamically adjusting the relationship between regular scale and input image size as required. To optimize resource utilization and enhance performance, additional modules, such as fused mobile inverted bottleneck convolution (Fused-MBConv), were integrated into EfficientNetV2. These modules ranged in depth from 2 to 6 and facilitated the development of deeper networks. Furthermore, EfficientNetV2 demonstrated superior adaptability across devices with varying processing complexities and input image sizes by outperforming several state-of-the-art models in common image classification tasks.

### 2.4. Model Fine-Tuning

In this experiment, five lightweight neural network models were selected, all of which used weights pre-trained on ImageNet. The advantage of pre-training with ImageNet was that these models could be used as general-purpose feature extractors for transfer learning for various computer vision tasks. Subsequently, the model was fine-tuned with the new wheat disease dataset to adapt to the recognition task.

**Figure 3:**
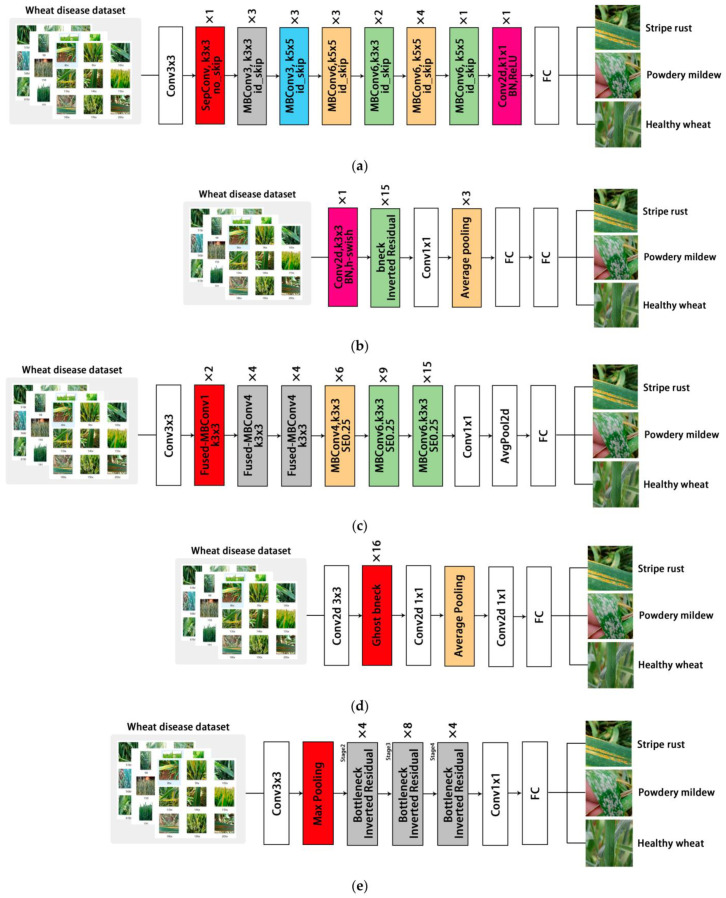
Diagrams of the five fine-tuned model structures: (**a**) MnasNet; (**b**) MobilieNetV3; (**c**) EffcientNetV3; (**d**) GhostNet; (**e**) ShuffleNetV2.

For the MobileNetV3, EfficientNetV2, and GhostNet models, we replaced only the last layer of the original model with a task-specific classification layer consisting of three output nodes. In MnasNet, we used a convolutional layer with a step size of 1, a convolution kernel size of 1 × 1, a two-dimensional batch normalization layer, and a ReLU activation function to replace the pooling layer before the full connected layer. The benefits of this were reduced information loss and improved model representation by introducing new nonlinear transformations, as well as reduced computation complexity. In ShuffleNetV2, the final pooling layer was removed to accommodate the output requirements of the new task. Detailed parameters after fine-tuning are shown in Table 2. Figure 3 shows the fine-tuned model structure diagram.

### 2.5. Model Optimization

#### 2.5.1. Learning Rate

The learning rate is a crucial hyper-parameter in deep learning, exerting a significant influence on model training [40]. It determines the magnitude of parameter updates made by the model in each iteration. Thus, the choice of the learning rate profoundly impacts the convergence. Moreover, an excessively high or low learning rate might pose challenges during training. If the learning rate is too high, it might hinder convergence or cause divergence as the model oscillates around the minimum loss value without reaching an optimal solution. Conversely, a low learning rate leads to slow convergence and a prolonged training duration while potentially trapping the model in local optima instead of finding global optima. Additionally, other hyper-parameters are influenced by the selection of learning rate. For instance, a smaller learning rate necessitates larger batch sizes to effectively utilize data for parameter adjustments.

In this study, three initial learning rates (0.05, 0.01, and 0.001) were employed alongside stepwise decay using step learning rate scheduler (StepLR) [41], which was a scheduling strategy that reduced the learning rate at specific epochs or steps to facilitate convergence and prevent overfitting. By multiplying the initial learning rate with a decay factor (gamma), better optimization results could be achieved after a certain number of epochs (step size). The StepLR formula was (1):

(1) $$lr=lr\times {gamma}^{{floor}^{\left(\frac{epoch}{step\_size}\right)}}$$

where *lr* is the learning rate, gamma is the decay factor, *epoch* is the number of rounds of the current training, *step size* is the interval indicating the adjustment of the learning rate, and *floor* is the function indicating the downward rounding.

#### 2.5.2. Optimizer

The optimizer plays a crucial role in training the neural network models, directly impacting both the training process and final performance. Stochastic gradient descent (SGD) [42], Adam [43], Adagrad [44], RMSprop [45], and AdaBelief [46] are commonly used optimization algorithms. Different optimization algorithms can have varying effects on the speed of updates, directionality, and convergence rate of parameters. Typically, optimizers involve the adjustment of the learning rate. For instance, the Adam optimizer dynamically modifies the learning rate as parameters update to provide adaptive learning rates. The gradients of the parameters are also computed and processed by the optimizer based on the loss function and are used for parameter adjustments during backpropagation. Various strategies, such as momentum and gradient clipping, are employed by different optimizers to manage gradients, which can enhance the training effectiveness and stability. In this study, Adam and SGD were chosen as optimizers.

#### 2.5.3. Warm-Up Training and Cosine Annealing

To achieve the global minimum of the loss function, it is crucial to reduce the learning rate during model training in order to bring the local minimum closer to the global minimum. Therefore, Abramson [47] initially proposed the cosine annealing (CA) algorithm, which utilized the cosine function for the gradual decline and subsequent acceleration and deceleration of the learning rate. In each epoch, the CA algorithm reduced the learning rate according to Equation (2):

(2) $$\eta_{t}=\eta_{{min}^{i}}+0.5\left(\eta_{{max}^{i}}-\eta_{{min}^{i}}\right)\left(1+\cos\frac{T_{cur}}{T_{i}}\right)$$

where *η_t_* is the learning rate, *η_min_* is the minimum learning rate set, *η_max_* is the maximum learning rate, *T_cur_* is the current iteration number, and *T_i_* is the maximum iteration number.

The weights of the model were randomly initialized using a learning rate warm-up at the beginning of training [48]. Initially, during the first few cycles, the learning rate might be relatively low, leading to stability in model performance. As a result, when stability was achieved, a preset learning rate was used for further training, which facilitated faster convergence and improved overall model performance.

### 2.6. Evaluation Indicators

The performance evaluation of an image classification model often relies on five commonly used indicators: accuracy, precision, recall, F1 score, and the confusion matrix heat map. Accuracy represents the percentage of correctly categorized examples out of the total. Precision measures a model’s ability to distinguish between positive and negative samples by calculating the ratio of correctly predicted positive samples. Recall quantifies the percentage of correctly predicted positive samples within a given sample set, indicating higher recall values for models that excel at identifying positive samples. The F1 score provided a balanced metric that considered both precision and recall. Lastly, the confusion matrix heat map visually presents predictions made by the classification model and facilitates the analysis of its strengths and weaknesses. Detailed calculation formulas for these indicators are provided as Equations (3)–(6).

(3) Recall=TP / (TP+FN)

(4) Precision=TP / (TP+FP) 

(5) Accuracy=(TP+TN) / (TP+TN+FP+FN)

(6) F1_score=2 ∗ (Precision ∗ Recall) / (Precision+Recall)

where *TP* is a positive sample of correct predictions, *FN* is a positive sample of incorrect predictions, *FP* is a negative sample of incorrect predictions, and *TN* is a negative sample of correct predictions.

### 2.7. Experimental Environment

The training process of the proposed model was run on the Windows 11 operating system and the PyTorch1.13.1 (GPU edition) framework. The software environments were CUDA11.6, CUDNN7.6, and Python3.8. The CPUs used to train the datasets were Intel(R) Core(TM) i5-11400H @ 2.70 GHz and 2.69 GHz 16G, and the GPU was an NVIDIA GeForce RTX 3050. The batch size was set to 16, and iterations were set to 50 echoes.

## 3. Results

### 3.1. The Learning Rate’s Effect on the Performance of Lightweight Models

**Figure 4:**
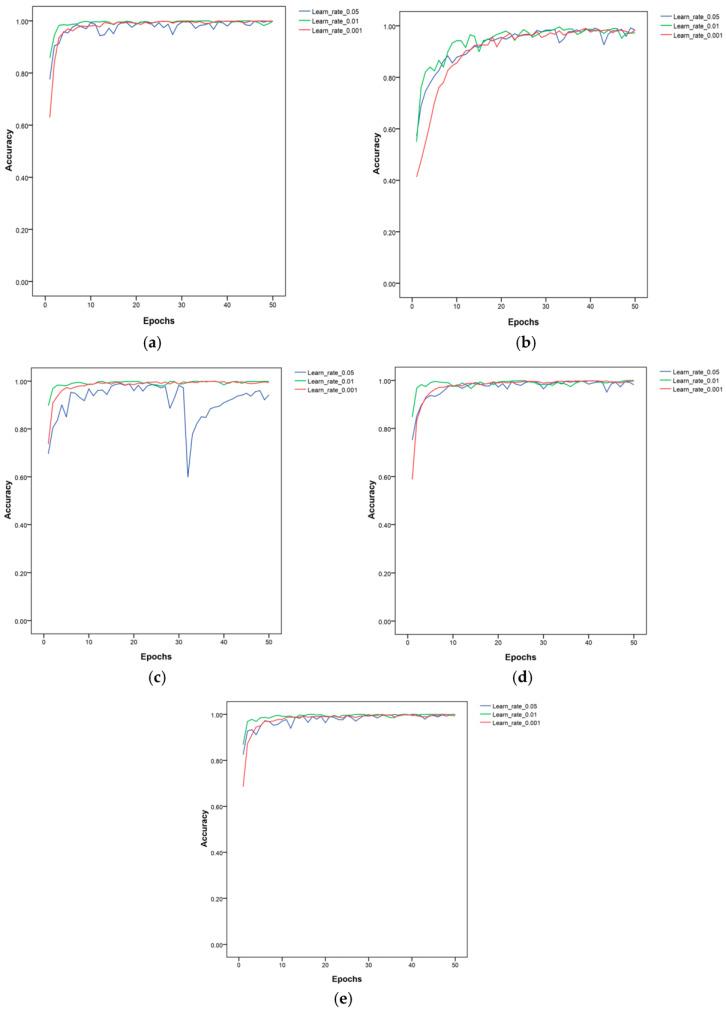
The accuracy of the five models at the 0.05, 0.01, and 0.001 learning rates: (**a**) EfficientNetV2; (**b**) GhostNet; (**c**) MobileNetV3; (**d**) MnasNet; (**e**) ShuffleNetV2.

**Figure 5:**
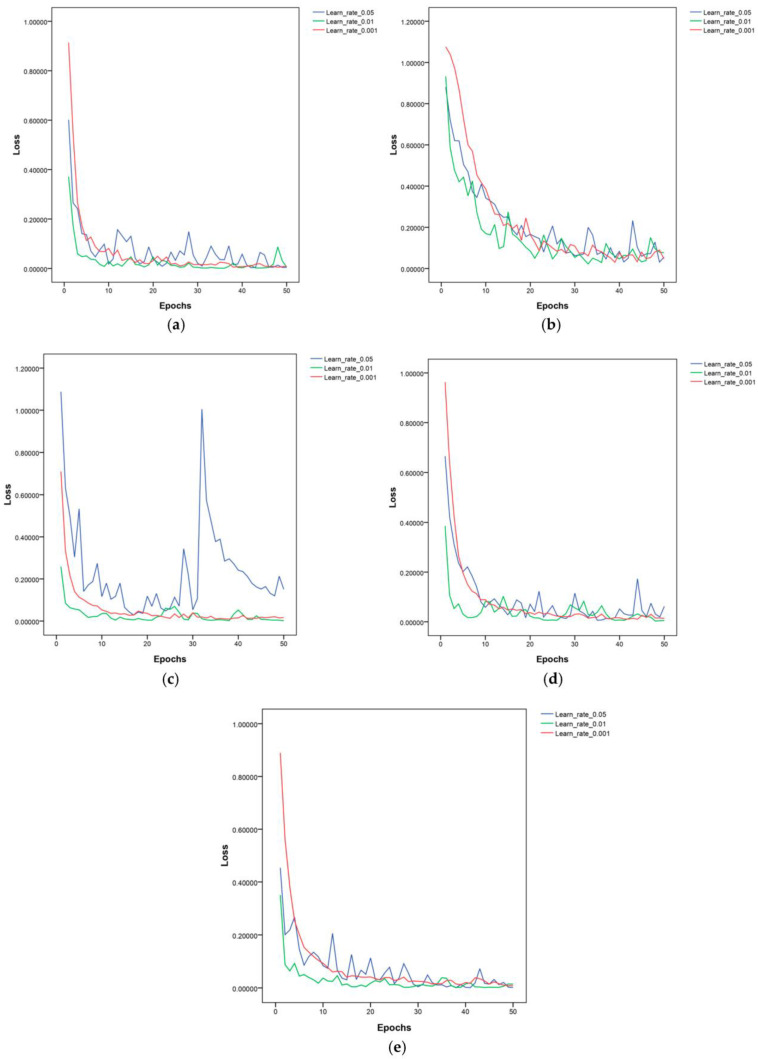
The loss values of the five models at the 0.05, 0.01, and 0.001 learning rates: (**a**) EfficientNetV2; (**b**) GhostNet; (**c**) MobileNetV3; (**d**) MnasNet; (**e**) ShuffleNetV2.

In order to evaluate the impact of the initial learning rate on the performance of the lightweight CNNs under the same experimental settings and parameters, three initial learning rates were set in this study along with a fixed learning rate to train the five lightweight CNNs. The accuracy and loss value were recorded. An SGD optimizer with the momentum parameter set to 0.8 was utilized to train the CNNs. The model’s parameters before and after fine-tuning are displayed in Table 2. Table 3 summarizes the average accuracy obtained by training five models three times at different learning rates. The details of the accuracy and loss values are shown in Figure 4 and Figure 5.

As shown in Table 3, the average accuracy of the five models on the training set was excellent, reaching more than 98%. However, the average accuracy on the test set was different. EfficientNetV2 had a better average accuracy regardless of the learning rate selected: all accuracy values were above 94%. When the learning rate was 0.001, the model had a better average accuracy of 97.52%. The average accuracy of MnasNet at a learning rate of 0.001 was the highest of the five models at 97.64%. The accuracy of other models decreased under different learning rate conditions. This indicated that the models were overfitted and had a weak generalization ability. Therefore, the MnasNet and EfficientNetV2 models were more suitable for identifying wheat diseases than the other three lightweight models. However, the MnasNet model had a smaller parameter at 19.09, which represented only a quarter of the parameters of the EfficientNetV2 model. The average test time of MnasNet was faster than that of the EfficientNetV2 by 6.04 s.

**Figure 6:**
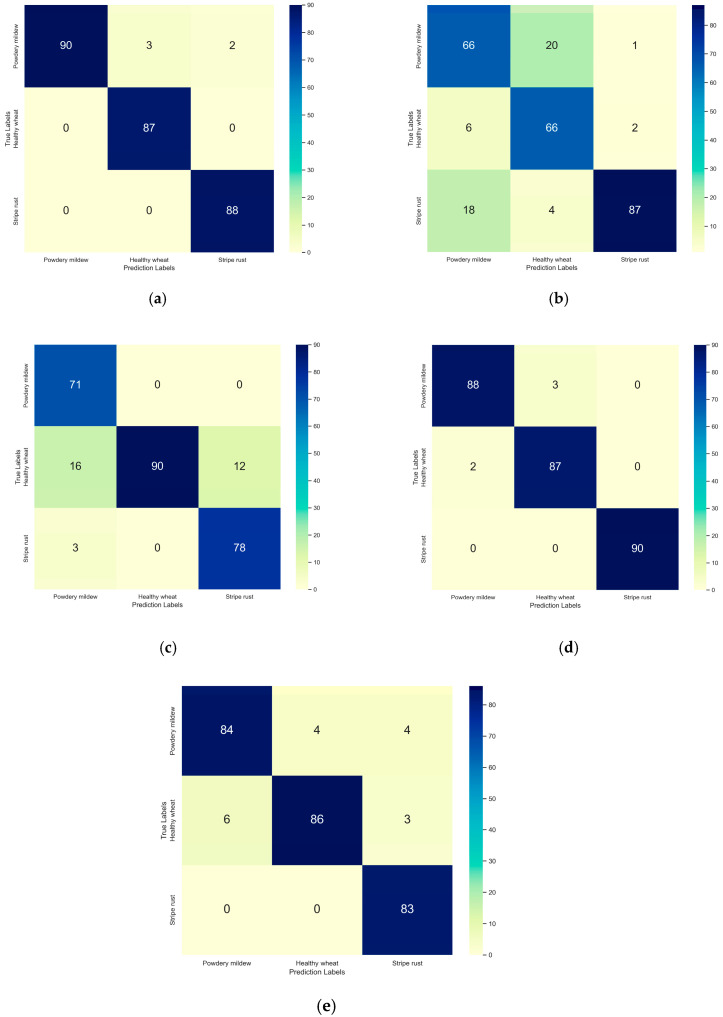
Confusion matrix analysis of five CNNs based on the test dataset: (**a**) EfficientNetV2; (**b**) GhostNet; (**c**) MobileNetV3; (**d**) MnasNet; (**e**) ShuffleNetV2. Note: the X-axis represents the predicted labels of wheat diseases, and the Y-axis represents the true labels of wheat diseases.

The confusion matrix of the five models with test datasets are shown in Figure 6. The EfficientNetV2, MnasNet, and ShuffleNetV2 models were able to better distinguish wheat stripe rust and powdery mildew from healthy leaves than the other models. The GhostNet model was able to distinguish wheat stripe rust well, but its performance in distinguishing wheat powdery mildew from healthy wheat leaves was not satisfactory. MobileNetV3 was good at identifying healthy leaves but poor at distinguishing between wheat powdery mildew and stripe rust.

### 3.2. Impact of Using Different Training Strategies on the Lightweight Models

**Figure 7:**
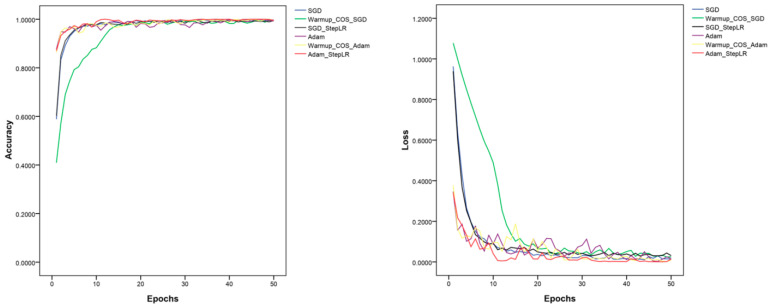
The accuracy and loss values under different training strategies.

Six strategies were used to train the five models: (1) Adam optimizer; (2) SGD optimizer; (3) Adam optimizer with learning rate decay (Adam + StepLR); (4) SGD optimizer with learning rate decay (SGD + StepLR); (5) Adam optimizer, warm-up, and cosine annealing; and (6) SGD optimizer, warm-up, and cosine annealing. The results obtained from training the five models three times each using different training strategies are shown in Table 4. MnasNet with an SGD optimizer with the learning rate decay exhibited the best recognition accuracy of 98.65%, which was 1.1% higher than seen in training with a fixed learning rate. MnasNet’s accuracy and loss values under the six strategies are shown in Figure 7. MobileNetV3 used an SGD optimizer with a learning rate decay, which improved accuracy by 1.24% more than if a fixed learning rate had been used. The other three models used an SGD optimizer with a fixed learning rate to achieve better results, and other training strategies caused the model accuracy to decrease by different degrees.

### 3.3. Model Testing

MnasNet trained using the SGD + StepLR strategy was utilized to identify the two wheat diseases, and the assessment indicators are provided in Table 5. When the F1 score was used as the final evaluation indicator, the model of wheat stripe rust had a slightly better classification performance of 99.45% than those of wheat powdery mildew and healthy wheat. The F1 scores of the MnasNet model used to recognize two wheat diseases demonstrated its excellent performance with values of around 99%, which could be attributed to the distinctive shapes and vibrant colors of the lesions of the typical symptoms.

**Figure 8:**
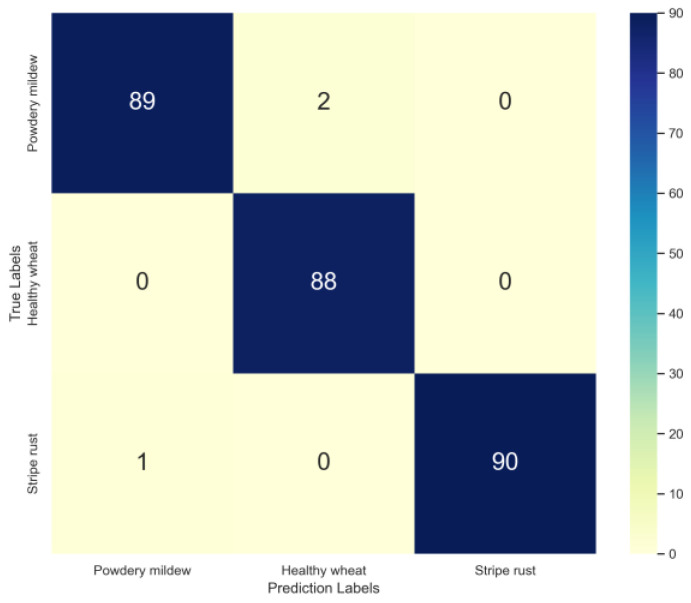
Confusion matrix of MnasNet.

As illustrated in Figure 8, the model’s classification results were used to visualize the confusion matrix. In the matrix, the value n_ij_ represents the number of times that the class i wheat disease was misidentified as class j. Higher values on the major diagonal suggest that the model had better recognition abilities.

## 4. Discussion

This study revealed that the performance of the model was significantly influenced by the learning strategies and hyper-parameters. Among these, the learning rate played a crucial role in model training, as it determined the network weights, which were adjusted based on the loss gradient. While opting for a low learning rate could ensure the avoidance of local minimums, it also led to longer convergence times, especially when encountering plateaus. Therefore, the optimal initial learning rate value for each model was found; exceeding this value prevented convergence, while falling below it resulted in slow convergence or a failure to learn [49]. In this study, five lightweight models were trained using three different learning rates, and it was found that the MnasNet model had the best performance with an accuracy of 97.64% while the EfficientNetV2 model’s accuracy was 97.52% when the learning rate was 0.001. Shahrabadi et al. [50] discovered that the convergence of the learning rate multi-model played an essential part in the training process. It was advantageous at a certain threshold to gradually reduce the learning rate. Models with high learning rates tended to exhibit higher loss scores and become unstable upon surpassing this threshold. Better results were obtained when the learning rates were 0.001 and 0.0001. In this study, different models were suitable for different learning rates; for example, the MnasNet model and the EfficientNetV2 model were more suitable for the 0.001 learning rate, and if the learning rate was too large, it might result in a poor training effect. This was consistent with Shahrabadi’s findings, which highlighted how excessive learning rates could lead to slower convergence and impede the obtaining of optimal solutions. However, the performances of the other three lightweight models were different at different learning rates, so it was important to explore the impact of the initial learning rate on the model.

The choice of optimizer depended on the specific situation, with each optimizer offering its own advantages. It was crucial to select the appropriate optimization algorithm based on the task requirements and data properties. In this study, Adam performed significantly worse than SGD, possibly due to its higher number of hyper-parameters. Moreover, Adam’s adaptive learning rate caused it to oscillate near the local optima. On the other hand, SGD only required the setting of a learning rate and had fewer hyper-parameters; however, this simplicity was also its drawback, as determining an optimal learning rate proved challenging, and manual adjustment was necessary. Different learning rates could lead to significant variations in training outcomes. Consequently, selecting an ideal initial learning rate played a critical role in determining the best training strategy. Compared to Adam, SGD occasionally jumped out of the local optima due to its random nature.

The GhostNet model exhibited a satisfactory performance in accuracy and loss values on the training data; however, its application to the test data revealed a significantly low accuracy, indicating overfitting. This issue might have arisen due to the model’s excessive complexity, limited training data size, and inappropriate feature selection as well as the fluctuating nature of the training data during the learning process. Therefore, L1 or L2 regularization should be included in future work. These techniques can effectively constrain model parameters and prevent them from becoming excessively large. Additionally, enhancing the generalization capacity of the model requires the evaluation of the performance of the model on diverse datasets using cross-validation.

Six training strategies were compared to evaluate their impacts on model performance. In the MnasNet and MobileNetV3 models, the learning rate decay strategy outperformed the fixed learning rate method. This is consistent with the results that a higher recognition accuracy can be achieved using the learning rate decay strategy [51]. The other three models were more suitable for fixed learning rates than decayed learning rates, which is consistent with Hideaki’s research [32]. The same model showed different effects under different training strategies, which resulted in significant differences. Therefore, the parameters of the training model should be selected according to the actual situation. Furthermore, it was discovered that the training effect of the SGD optimizer was superior to that of the Adam optimizer, which was consistent with the conclusion reached by Wang et al. [52], who demonstrated that even though Adam has a faster convergence speed than SGD, the model could not achieve higher accuracy, indicating that Adam optimizer’s generalization ability was poor. The Adam optimizer was not particularly successful at identifying the flat minimum, which was critical for the generalization ability. Therefore, SGD optimizers are still in use today. The five models displayed poor performances when using the warming-up and cosine annealing algorithms. Thus, automatically adjusting the learning rate was not always applicable in training models. It was therefore important to explore the most appropriate training method in this study.

In this study, only two wheat diseases were distinguished from healthy wheat leaves in the recognition models. The fine-tuned model has not been tested on other datasets. However, for the tested models, MnasNet has fewer parameters and a fast detection speed, which makes it suitable for use on mobile devices.

In future, we will: (1) increase the types and quantity of wheat diseases in the dataset, (2) continue to improve and optimize the model and develop a disease identification software, and (3) test the optimized and improved model on other datasets.

## 5. Conclusions

This study developed five lightweight models for the recognition of wheat stripe rust, powdery mildew, and healthy wheat leaves using three different learning rates based on transfer learning. Of these models, MnasNet and EfficientNetV2 were found to display an excellent performance using an initial learning rate of 0.001, with accuracies of 97.64% and 97.52%, respectively. In addition, the accuracy of the MnasNet model was higher than that of EfficientNetV2 by 0.12%, and the model size was smaller than that of EfficientNetV2 by 75%, which demonstrated that the MnasNet model is suitable for deployment on mobile devices. The MnasNet model adopted the SGD (with momentum) optimizer and learning rate attenuation strategy, and its accuracy reached 98.65%, which was 1.1% higher than that of the fixed learning rate model and yielded better results. Thus, this allows MnasNet to be effectively and practically utilized as a robust lightweight convolutional neural network on mobile devices.

## Figures and Tables

**Table 1:** Datasets of wheat leaf disease.

Type	Training Set	Validation Set	Test Set
Stripe rust	630	180	90
Powdery mildew	630	180	90
Healthy wheat	630	180	90
Total samples	1890	540	270

**Table 2:** Parameters of the five lightweight CNN models.

Models	Layer	Original Parameters (M)	Fine-Tuned Parameters (M)
MobileNetV3-large	191	20.91	16.04
ShuffleNetV2_x2.0	167	28.21	20.41
GhostNetV1	282	19.77	14.89
EfficientNetV2-s	550	81.86	76.98
MnasNet1_3	157	23.96	19.09

**Table 3:** Accuracy and test times of different models at different learning rates.

Models	Learning Rate	Train Accuracy (%)	Average Train Accuracy (%)	Test Accuracy (%)	Average Test Accuracy (%)	Test Time (s)
MobileNetV3-large	0.05	99.94	99.39	92.96	87.52	14.75
		98.94		91.85		
		99.31		77.77		
	0.01	100.00	100.00	93.70	85.31	15.01
		100.00		89.25		
		100.00		73.00		
	0.001	99.94	99.92	87.77	87.89	15.52
		99.89		88.51		
		99.94		87.40		
ShuffleNetV2_x2.0	0.05	99.94	99.92	77.77	77.88	15.68
		100		85.15		
		99.84		70.74		
	0.01	100.00	99.96	93.70	91.48	15.92
		99.89		93.33		
		100.00		87.04		
	0.001	100.00	100.00	84.07	80.98	15.33
		100.00		72.96		
		100.00		85.92		
GhostNetV1	0.05	99.15	98.89	71.85	74.19	15.43
		99.15		81.11		
		98.37		69.62		
	0.01	99.57	99.53	67.03	66.91	15.26
		99.73		73.33		
		99.31		60.37		
	0.001	98.84	98.89	65.92	62.96	15.93
		98.84		60.74		
		99.00		62.22		
EfficientNetV2-s	0.05	100.00	100.00	95.18	94.19	21.01
		100.00		94.07		
		100.00		93.33		
	0.01	100.00	100.00	92.59	94.93	21.68
		100.00		95.18		
		100.00		97.03		
	0.001	100.00	99.94	97.77	97.52	21.36
		99.89		98.14		
		99.94		96.66		
MnasNet1_3	0.05	99.84	99.78	55.18	62.34	15.39
		99.78		63.33		
		99.73		68.51		
	0.01	99.89	99.87	87.03	78.26	15.54
		100		83.70		
		100		64.07		
	0.001	99.89	99.85	97.77	97.64	15.01
		99.84		98.14		
		99.84		97.03		

**Table 4:** The average accuracy of the five models under different training strategies.

Model	Training Strategy	Test Accuracy	Average Test Accuracy
MobileNetV3	SGD	87.77	87.89
		88.51	
		87.40	
	SGD + StepLR	86.30	89.13
		85.93	
		91.85	
	Warm-up + cosine annealing + SGD	86.30	86.79
		85.93	
		88.15	
	Adam	88.52	85.06
		80.00	
		86.67	
	Adam + StepLR	85.19	87.53
		90.74	
		86.67	
	Warm-up + cosine annealing + Adam	85.93	87.53
		87.41	
		89.26	
MnasNet	SGD	87.77	97.64
		88.51	
		87.40	
	SGD + StepLR	98.15	98.65
		98.89	
		98.89	
	Warm-up + cosine annealing + SGD	87.78	94.19
		98.15	
		96.67	
	Adam	60.00	67.03
		61.11	
		80.00	
	Adam + StepLR	96.30	95.67
		95.19	
		95.56	
	Warm-up + cosine annealing + Adam	79.26	85.80
		86.30	
		91.85	
EfficientNetV2	SGD	97.77	97.52
		98.14	
		96.66	
	SGD + StepLR	95.93	97.04
		97.41	
		97.78	
	Warm-up + cosine annealing + SGD	91.11	94.20
		96.67	
		94.81	
	Adam	90.37	85.06
		85.56	
		79.26	
	Adam + StepLR	91.11	84.69
		83.33	
		79.63	
	Warm-up + cosine annealing + Adam	81.11	84.07
		87.78	
		83.33	
GhostNet	SGD	71.85	74.19
		81.11	
		69.62	
	SGD + StepLR	61.48	70.99
		73.33	
		78.15	
	Warm-up + cosine annealing + SGD	71.48	62.84
		61.11	
		55.93	
	Adam	56.67	48.89
		32.59	
		57.41	
	Adam + StepLR	33.70	31.98
		27.41	
		34.81	
	Warm-up + cosine annealing + Adam	49.26	44.07
		33.33	
		49.63	
ShuffleNetV2	SGD	93.70	91.48
		93.33	
		87.40	
	SGD + StepLR	81.11	79.51
		72.96	
		84.44	
	Warm-up + cosine annealing + SGD	92.96	88.52
		86.67	
		85.93	
	Adam	78.15	69.88
		61.11	
		70.37	
	Adam + StepLR	84.07	77.04
		78.89	
		68.15	
	Warm-up + cosine annealing + Adam	65.93	64.69
		58.52	
		69.63	

**Table 5:** Classification results of the model.

Diseases of Wheat	Accuracy (%)	Precision (%)	Recall (%)	F1 Score (%)
Powdery mildew	98.89	97.80	98.89	98.34
Healthy wheat	99.28	100.00	97.78	98.88
Stripe rust	99.63	98.90	100.00	99.45

## Data Availability

The data are contained within the article.
